# Personalized medicine in the dish to prevent calcium leak associated with short-coupled polymorphic ventricular tachycardia in patient-derived cardiomyocytes

**DOI:** 10.1186/s13287-023-03502-5

**Published:** 2023-09-23

**Authors:** Yvonne Sleiman, Steven Reiken, Azzouz Charrabi, Fabrice Jaffré, Leah R. Sittenfeld, Jean-Luc Pasquié, Sarah Colombani, Bruce B. Lerman, Shuibing Chen, Andrew R. Marks, Jim W. Cheung, Todd Evans, Alain Lacampagne, Albano C. Meli

**Affiliations:** 1 PhyMedExp, University of Montpellier, CNRS, INSERM, Montpellier , France; 2 Department of Physiology and Cellular Biophysics, Clyde and Helen Wu Center for Molecular Cardiology, Columbia University Vagelos College of Physicians and Surgeons, New York, NY USA; 3 Department of Surgery, Weill Cornell Medical College, New York, NY USA; 4 Department of Cardiology, CHRU of Montpellier, Montpellier, France; 5 Division of Cardiology, Weill Cornell Medical College, New York, NY USA; 6 CNRS, INSERM, Montpellier Organoid Platform, Biocampus, University of Montpellier, Montpellier, France

**Keywords:** Short-coupled PMVT, Isogenic control, Cardiac ryanodine receptor, hiPSC-derived cardiomyocytes, Drug screening, Calcium handling, Contractile properties, Post-translational modifications

## Abstract

**Background:**

Polymorphic ventricular tachycardia (PMVT) is a rare genetic disease associated with structurally normal hearts which in 8% of cases can lead to sudden cardiac death, typically exercise-induced. We previously showed a link between the RyR2-H29D mutation and a clinical phenotype of short-coupled PMVT at rest using patient-specific hiPSC-derived cardiomyocytes (hiPSC-CMs). In the present study, we evaluated the effects of clinical and experimental anti-arrhythmic drugs on the intracellular Ca^2+^ handling, contractile and molecular properties in PMVT hiPSC-CMs in order to model a personalized medicine approach in vitro.

**Methods:**

Previously, a blood sample from a patient carrying the RyR2-H29D mutation was collected and reprogrammed into several clones of RyR2-H29D hiPSCs, and in addition we generated an isogenic control by reverting the RyR2-H29D mutation using CRIPSR/Cas9 technology. Here, we tested 4 drugs with anti-arrhythmic properties: propranolol, verapamil, flecainide, and the Rycal S107. We performed fluorescence confocal microscopy, video-image-based analyses and biochemical analyses to investigate the impact of these drugs on the functional and molecular features of the PMVT RyR2-H29D hiPSC-CMs.

**Results:**

The voltage-dependent Ca^2+^ channel inhibitor verapamil did not prevent the aberrant release of sarcoplasmic reticulum (SR) Ca^2+^ in the RyR2-H29D hiPSC-CMs, whereas it was prevented by S107, flecainide or propranolol. Cardiac tissue comprised of RyR2-H29D hiPSC-CMs exhibited aberrant contractile properties that were largely prevented by S107, flecainide and propranolol. These 3 drugs also recovered synchronous contraction in RyR2-H29D cardiac tissue, while verapamil did not. At the biochemical level, S107 was the only drug able to restore calstabin2 binding to RyR2 as observed in the isogenic control.

**Conclusions:**

By testing 4 drugs on patient-specific PMVT hiPSC-CMs, we concluded that S107 and flecainide are the most potent molecules in terms of preventing the abnormal SR Ca^2+^ release and contractile properties in RyR2-H29D hiPSC-CMs, whereas the effect of propranolol is partial, and verapamil appears ineffective. In contrast with the 3 other drugs, S107 was able to prevent a major post-translational modification of RyR2-H29D mutant channels, the loss of calstabin2 binding to RyR2. Using patient-specific hiPSC and CRISPR/Cas9 technologies, we showed that S107 is the most efficient in vitro candidate for treating the short-coupled PMVT at rest.

**Supplementary Information:**

The online version contains supplementary material available at 10.1186/s13287-023-03502-5.

## Background

Cardiac channelopathies are most often caused by mutations in genes coding for ion channels. Catecholaminergic polymorphic ventricular tachycardia (CPVT) is a channelopathy characterized by polymorphic ventricular tachycardias (PMVT) triggered by catecholamines released during exercise, stress or emotion, that may lead to sudden death, mostly in children, teenagers and young adults with structurally normal hearts. Most of them are linked to cardiac ryanodine receptor type 2 (RyR2) mutations. During cardiac excitation–contraction coupling (ECC), the cardiac ryanodine receptor/calcium (Ca^2+^) release channel plays a major role by releasing a large amount of Ca^2+^ preceding and initiating the contraction [1, 2]. Single-point mutations in RyR2 have been reported to induce arrhythmogenic disorders including catecholaminergic PMVT (CPVT) [3] and arrhythmogenic right ventricular dysplasia (ARVC/D2) under stress conditions [4, 5].

However, we also identified a link between a novel RyR2 mutation (RyR2-H29D) and short-coupled PMVT among family members who experienced syncope and a short-coupled PMVT at rest and not with exercise, consistent with a clinical phenotype that was distinct from CPVT [6]. Using a heterologous expression system, we showed that the RyR2-H29D mutation, although not located in any RyR2 hot-spot regions, causes a gain-of-function associated with SR Ca^2+^ leak through RyR2 at diastolic Ca^2+^ levels under non-stress conditions [6]. We then modeled the PMVT syndrome in the dish using patient-specific derived cardiomyocytes carrying RyR2-H29D or the gene corrected by CRISPR/Cas9 technology (i.e., patient-specific isogenic control). We demonstrated that the RyR2-H29D mutation induces an abnormal intracellular Ca^2+^ homeostasis, abnormal mechanical and electrical properties and RyR2 post-translational remodeling including PKA-phosphorylation, oxidation, S-nitrosylation and calstabin2 depletion. These abnormal features were abolished in the isogenic control hiPSC-CMs, proving that RyR2 dysfunction can be associated with short-coupled PMVT occurring at rest with a phenotype that is distinct from CPVT [7]. In the present study, we tested 4 drugs with anti-arrhythmic effects, which include three drugs that are currently clinically prescribed for PMVT. Specifically, we examined propranolol (β-blocker classified as a class II antiarrhythmic), verapamil (voltage-dependent Ca^2+^ channel inhibitor, class IV antiarrhythmic) [8, 9], flecainide (voltage-dependent Na^+^ channel and RyR2 inhibitor, class IC antiarrhythmic drug) [10] and S107, an experimental Rycal compound known to stabilize the closed conformation of RyR2 [11–18]. We performed biochemistry of RyR2, fluorescence confocal microscopy and video-image-based analysis to investigate the effect of these 4 drugs on the molecular and functional features of PMVT (RyR2-H29D) and isogenic control hiPSC-CMs.

## Materials and methods

### Cell lines

The hiPSC lines were generated from a blood sample of a 32-year-old female patient exhibiting the short-coupled PMVT at rest and carrying the RyR2-H29D mutation, as previously characterized [6] with the consent of the index patient and following the approval by the Institutional Review Board of Weill Cornell Medicine (NY, USA). As previously described, the RyR2-H29D mutation was corrected using CRISP/Cas9 technology thus generating an isogenic control hiPSC line (PMVT-29-corrected) [7]. The hiPSC lines were maintained on hES-qualified Matrigel (Corning, 354,277) at standard conditions (21% O_2_, 5% CO_2_ and 37 °C) and were enzymatically dissociated using TrypLE enzyme (Gibco, ref: 12,604–013) and passaged every 4 days. As 2 independent clones of PMVT RyR2-H29D hiPSC (C1, C3) tested in a previous study [7] exhibited identical properties, here we focused on the C1 clone only.

### Cardiac differentiation

The 2D-sandwich based protocol, was used to differentiate the hiPSC lines to the cardiac lineage in standard conditions as previously described [7, 11, 19]. Briefly, mesoderm was induced through the activation of the Wnt/β-catenin pathway via 6 µM CHIR99021 (Calbiochem, ref: 3,615,715). The cardiac progenitor formation was induced via the inhibition of the Wnt/β-catenin pathway by 2 µM Wnt inhibitor C59 (Wnt C59) (Tocris, ref: 5148/10). To obtain a pure population of hiPSC-CMs, we treated the dish using SILAC medium supplemented with 4 mM Na^+^-lactate in absence of glucose and pyruvate from day 11 to 16 as published [20]. To obtain a more mature phenotype the commercially-available Pluricyte medium (Ncardia, ref: PM-2100) was employed for 10 days. The hiPSC-CMs were maintained in culture until day 60 of differentiation as previously described [7].

### Immunoprecipitation and immunoblot analyses

The 2D cardiac sheets were mechanically dissected using a needle and homogenized with a lysis buffer composed of 35 mM NaF, 50 mM Tris maleate pH 6.8, 1 mM Na_3_VO_4_ and protease inhibitors for 5 min on ice. Cell lysate was sonicated 5 times for 15 s each. The extracted samples were then centrifuged for 20 min at 8000 g at 4 °C. The whole lysate was then collected and used for protein concentration assay, Western blot and immunoprecipitation as previously described [7]. Briefly, the immunoprecipitation of RyR2 channels was performed using an anti-RyR antibody (homemade antibody: rabbit 5029 y2) in RIPA buffer composed of 10 mM Tris–HCl pH 7.4, 150 mM NaCl, 5 mM NaF, 1 mM Na_3_VO_4_, 1% Triton-X100, and protease inhibitors. The protein A sepharose beads (GE Healthcare, ref: 17–5280-01) were used for purifying the immune samples. 4–20% SDS-PAGE gradient gel was used to separate the proteins which were incubated with the following primary antibodies: rabbit 5029 Y2 anti-RyR2 (1:5000), anti-phospho-RyR2-pSer2809 (homemade antibody: polyclonal rabbit CRTRRI- (pS)-QTSQ, 1:1000), anti-RyR2-pSer2815 (homemade antibody: polyclonal rabbit CSQTSQV-(pS)-VD), anti-DNP antibody (Millipore,1:2000), anti-Cys-NO (Sigma-Aldrich, 1:1000) and mouse anti-FKBP12.6 (Santa Cruz, ref: 376,135, 1:1000). The Odyssey system (LI-COR) was used to develop the immunoblots with IR labeled secondary antibodies (1:30,000 dilution) for 1 h at room temperature.

### Monolayer dissociation of hiPSC-CMs

To obtain isolated single hiPSC-CMs for intracellular Ca^2+^ imaging acquisition, 2D cardiac sheets were dissociated using TrypLE enzyme as previously described [7]. Briefly, the monolayer cells were washed twice with Ca^2+^- and Mg^2+^-free PBS (Sigma, ref: D8537) and then incubated with pre-warmed TrypLE for 10 min at 37 °C. The enzymatic activity of TrypLE was stopped using RPMI 1640-B27 (Gibco, ref: 21,875–034). The remaining cell clumps were filtered using a 37-µm reversible strainer (Stemcell Technologies, ref: 27,215). The whole lysate was then centrifuged for 5 min at 200 g, and the cell pellet resuspended with RPMI 1640-B27 containing ROCK inhibitor (Miltenyi Biotec, ref: 130–106-538). The cells were then plated on MatTek Corporation dishes pre-coated with hES-qualified Matrigel in RPMI 1640-B27 medium for Ca^2+^ imaging acquisition.

### Measurement of cytosolic Ca^2+^ variation under fluorescence confocal microscopy

The intracellular Ca^2+^ variations in hiPSC-derived cardiomyocytes were measured and analyzed using 60-day-old single cardiomyocytes that were enzymatically dissociated from monolayer cells as previously described [7]. The hiPSC-CMs were incubated with 1.5 µM of non-ratiometric Fluo-4 AM Ca^2+^ indicator (Molecular Probes) for 15 min in the dark which was excited at 488 nm. Using an inverted confocal fluorescence microscope equipped with Zeiss LSM780 confocal and 63 × lens (oil immersion, numerical aperture, N.A. = 1.4), images were recorded in line-scan mode (i.e. , x-t mode, 1.53 ms per line; 512 pixels × 5,000 lines) using Zen (Zeiss) at RT. The 4 drugs were tested upon 1 Hz pacing (20 V and 3 ms duration using S88 dual output square Pulse stimulator from Grass Instruments) with the following final concentration: 3 µM of propranolol, 10 nM of verapamil, 5 µM of flecainide and 5 µM S107 [13, 21]. The propranolol, verapamil, flecainide and S107 were incubated 10 min prior to acquisition. All data were extracted using Zen software. The different parameters collected were analyzed using the Peak-inspector algorithm made via Python 3 (https://www.python.org/download/releases/3.0/) as previously done [7, 11].

### Measurement of the contractile properties by video-edge capture

The contractile properties of the monolayer cardiac tissue were measured in 6 well plates containing the beating hiPSC-CMs. The cells were allowed to stabilize in the thermostatic chamber at 21% O_2_ and 5% CO_2_ at 37 °C prior to any recording using an inverted Zeiss observer 7 microscope equipped with a 20 × objective (N.A. = 0.4). Different spontaneous beating regions were recorded with an Orca Falsh4 camera (Hamamatsu) with an imaging frequency of 33 ms, at 16-bit depth and a duration of 25 s per position. 4 drugs were tested in the video-edge experiments: 3 µM propranolol, 100 nM verapamil, 5 µM flecainide and 5 µM S107. Importantly, the acquisitions were first performed without drugs. Then, each drug was incubated 20 min prior to further acquisition. It should be noted that the same regions chosen without drugs were used for the acquisition with drugs in order to obtain the direct effect of the drug on the same region (paired experiments). The contractile parameters were analyzed using a patented custom-made video analysis software (MATLAB) as we have previously published [7, 22, 23].

### Statistical analysis

Statistical analysis was performed using the GraphPad Prism software (version 9). Normality was tested using the Shapiro–Wilk test. A paired t test was used to compare 2 paired groups with parametric distribution. A Wilcoxon test was performed for comparing 2 paired experiments with nonparametric distribution. An unpaired t test was used to compare 2 independent groups with parametric distribution. A Mann–Whitney test was performed for comparing 2 independent groups with nonparametric distribution. ANOVA test was performed for comparing multiple independent groups with parametric distribution. Kruskal–Wallis test was performed for comparing multiple independent groups with nonparametric distribution. All data are presented as mean ± SEM. A value of *p* < 0.05 was considered significant. *, *p* < 0.05, **, *p* < 0.01, ***, *p* < 0.001 unless otherwise specified.

## Results

### Only S107 prevents calstabin2 depletion from the RyR2 macromolecular complex

We previously demonstrated that RyR2-H29D undergoes post-translational modifications including PKA-phosphorylation, S-nitrosylation, oxidation and depletion of Calstabin2 under non-stress conditions [6, 7]. We therefore selected drugs that we hypothesized could modulate RyR2 macromolecular remodeling for study. Specifically, we selected propranolol (non-selective β-blocker) that is prescribed to treat CPVT [24, 25], verapamil (a dihydropyridine receptor inhibitor [DPHR]) that has been proposed to be a first-line treatment of short-coupled torsades de pointes [9], flecainide (Na^+^ channel inhibitor) known to be effective in treatment of CPVT [11] and S107 (stabilizer of the RyR2 closed state conformation) [21, 26].

**Fig. 1:**
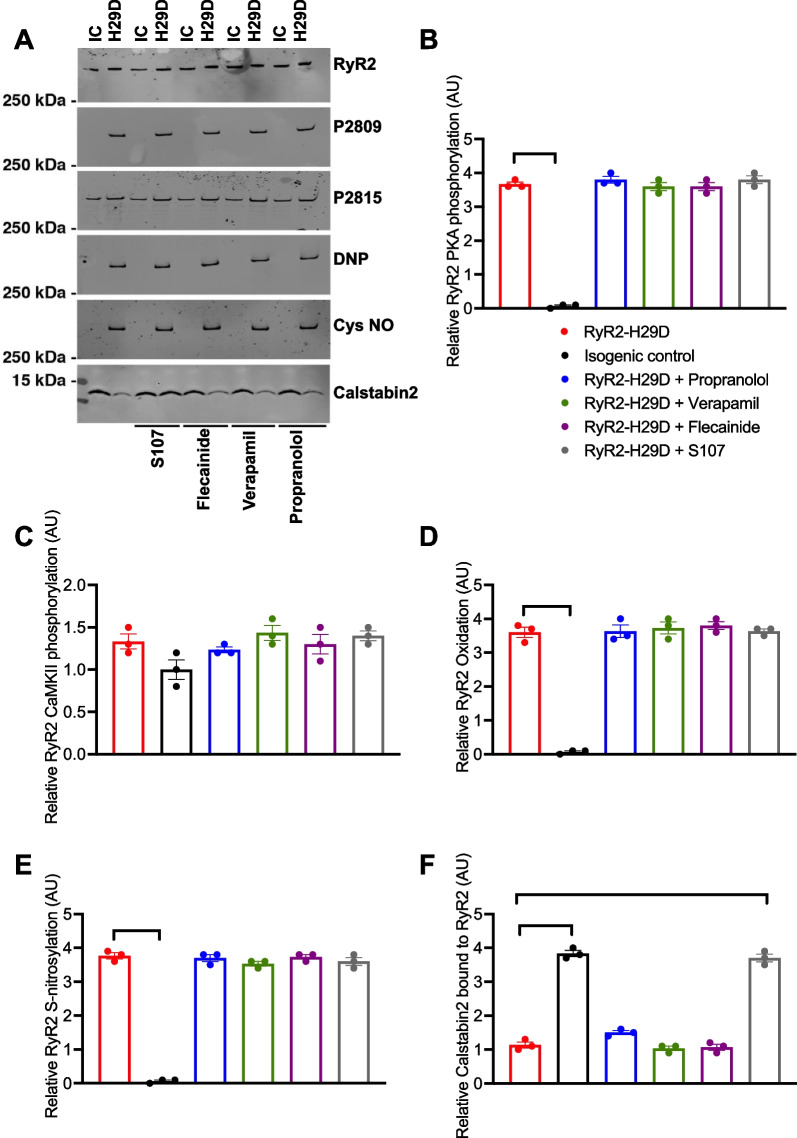
S107 prevents calstabin2 depletion in RyR2-H29D hiPSC-CMs. **A** Immunoblots of the RyR2 co-immunoprecipitation showing the level of PKA and CaMKII-phosphorylation at Ser2809 (P2809) and Ser2815 (P2815), respectively, oxidation of cysteine (DNP), S-nitrosylation of cysteine (Cys-NO) and calstabin2 (FKBP12.6) binding in isogenic control and treated and untreated RyR2-H29D hiPSC-CMs with propranolol (3 µM), verapamil (10 nM), flecainide (5 µM) and S107 (5 µM).** B** Relative RyR2 PKA-phosphorylation level at Ser2809 in isogenic control and treated and untreated RyR2-H29D hiPSC-CMs with the 4 drugs. **C** Relative RyR2 CaMKII-phosphorylation level at Ser2815 in isogenic control and treated and untreated RyR2-H29D hiPSC-CMs with the 4 drugs. **D** Relative RyR2 oxidation level in isogenic control and treated and untreated RyR2-H29D hiPSC-CMs with the 4 drugs. **E** Relative RyR2 S-nitrosylation level in isogenic control and treated and untreated RyR2-H29D hiPSC-CMs with the 4 drugs.** F** Relative calstabin2 amount bound to RyR2 in isogenic control and treated and untreated RyR2-H29D hiPSC-CMs with the 4 drugs. The number of experiments is based on 3 independent biological replicates. Significance was calculated by Kruskal–Wallis test. Data are presented as mean ± SEM. **, *p* < 0.01. *IC*: Isogenic control and *H29D*: RyR2-H29D

RyR2 co-immunoprecipitation revealed that PKA- and CaMKII-phosphorylation (at Ser2809 and Ser2815, respectively) as well as S-nitrosylation and oxidation of RyR2-H29D were unchanged by treatment of the 4 drugs (Fig. 1A–E). Only S107 prevented the loss of calstabin2 binding to RyR2, restoring this to a level similar as the isogenic control (1.13 ± 0.08 for the RyR2-H29D vs. 3.70 ± 0.11 for RyR2-H29D + S107, *p* < 0.01 vs. 3.83 ± 0.08 for isogenic control, *p* < 0.01) (Fig. 1A and F). None of the drugs affected the RyR2 remodeling in the isogenic control hiPSC-CMs (Additional file 1: Fig. S1A–E). Full-length blots/gels are presented in Additional file 1: Fig. S2.

### Flecainide and S107 prevent the aberrant release of Ca^2+^ in RyR2-H29D hiPSC-CMs

**Fig. 2:**
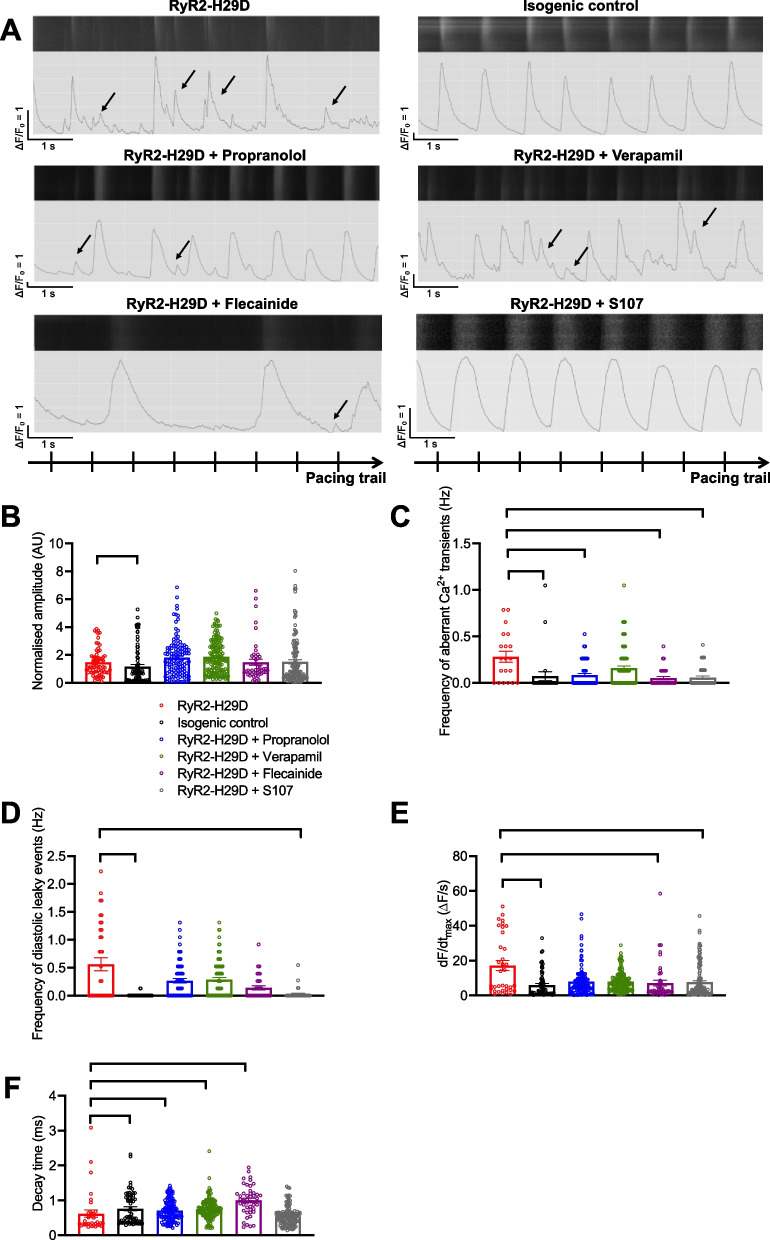
S107 is the most effective drug to prevent the aberrant Ca^2+^ release in RyR2-H29D hiPSC-CMs. **A** Display of original line-scan images of Ca^2+^ transients and corresponding tracings and pacing trail in RyR2-H29D hiPSC-CMs, isogenic control, RyR2-H29D hiPSC-CMs treated with propranolol (3 µM for 10 min), verapamil (10 nM for 10 min), flecainide (5 µM for 10 min) and S107 (5 µM for 10 min) under 1 Hz pacing (20 V and 5 ms duration). Additional and aberrant Ca^2+^ release events are shown with the arrows. **B** Normalized Ca^2+^-transient amplitude in RyR2-H29D hiPSC-CMs (red bars and red dots plot), isogenic control (black bars and black dots plot), RyR2-H29D treated with propranolol (blue bars and blue dots plot), verapamil (green bars and green dots plot), flecainide (purple bars and purple dots plot) and S107 (gray bars and gray dots plot) under 1 Hz pacing. **C** Frequency of occurrence of aberrant Ca^2+^-transients in RyR2-H29D, isogenic control, RyR2-H29D treated with propranolol, verapamil, flecainide and S107.** D** Frequency of occurrence of diastolic leaky events in RyR2-H29D, isogenic control, RyR2-H29D treated with propranolol, verapamil, flecainide and S107.** E** Rate of RyR2 Ca^2+^ release (dF/dt_max_ in ΔF/s) in RyR2-H29D, isogenic control, RyR2-H29D treated with propranolol, verapamil, flecainide and S107. **(F)** Decay time in RyR2-H29D, isogenic control, RyR2-H29D treated with propranolol, verapamil, flecainide and S107. The number of experiments is based on 3 independent biological replicates. The number of experiments varies from 20 to 152 cells for each scatter plot. Significance was calculated by Kruskal–Wallis test. Data are presented as mean ± SEM. *, *p* < 0.05, **, *p* < 0.01, ***, *p* < 0.001

We then evaluated whether any of the 4 drugs could prevent the intracellular Ca^2+^ mishandling previously described in RyR2-H29D hiPSC-CMs [6, 7]. Flecainide [10, 27, 28] and particularly S107 [12, 15, 16] were shown experimentally to prevent the aberrant release of Ca^2+^ in CPVT murine and cellular human models. When applied on RyR2-H29D hiPSC-CMs, none of the drugs changed the Ca^2+^ transient amplitude in RyR2-H29D hiPSC-CMs at 1 Hz pacing (Fig. 2A and B). Propranolol, flecainide and S107 decreased the occurrence of aberrant Ca^2+^ transients while verapamil treatment did not (Fig. 2A and C). Of the 4 drugs, only S107 treatment decreased the frequency of diastolic SR Ca^2+^ leaky events (frequency of occurrence of 0.56 ± 0.11 Hz for the RyR2-H29D vs. 0.02 ± 0.01 Hz for RyR2-H29D + S107, *p* < 0.01) (Fig. 2A and D). Applying flecainide or S107 decreased the RyR2 Ca^2+^-release velocity (rate of Ca^2+^ release of 17.20 ± 2.83 ∆F/s for the RyR2-H29D vs. 7.10 ± 1.61 ∆F /s for the RyR2-H29D + flecainide, and vs. 7.66 ± 0.83 ∆F /s for the RyR2-H29D + S107, *p* < 0.01) in contrast with the other drugs (Fig. 2A and E). The decay time increased in RyR2-H29D hiPSC-CMs upon treatment with the 3 drugs, in particular with flecainide (0.61 ± 0.10 ms for the RyR2-H29D vs. 1.00 ± 0.05 ms for RyR2-H29D + flecainide, *p* < 0.001) but not with S107 treatment (Fig. 2A and F). Overall, verapamil treatment only induced a decay time increase (0.61 ± 0.10 ms for the RyR2-H29D vs. 0.75 ± 0.02 ms for RyR2-H29D + verapamil, *p* < 0.01) (Fig. 2A and F). Unexpectedly, verapamil and flecainide caused aberrant release of Ca^2+^ in the isogenic control hiPSC-CMs (Additional file 1: Fig. S3). In summary, Table 1 provides an overview of the effects of the four drugs on SR Ca^2+^ handling in RyR2-H29D hiPSC-CMs.

**Table 1:** Summary and grading of the effects of propranolol, verapamil, flecainide and S107 on the SR Ca^2+^ handling, contractile properties and RyR2 remodeling in RyR2-H29D (PMVT) hiPSC-CMs

		Propranolol	Verapamil	Flecainide	S107
		RyR2-H29D	RyR2-H29D	RyR2-H29D	RyR2-H29D
SR Ca^2+^ handling properties	Normalized Amplitude	0	0	0	0
	Aberrant Ca^2+^ transients	+ 1	0	+ 1	+ 1
	Diastolic leaky events	0	0	0	+ 1
	Rate of RyR2 Ca^2+^ release	0	0	+ 1	+ 1
	Decay time	+ 1	+ 1	+ 1	0
Contractile properties	Beat rate	− 1	− 1	− 1	− 1
	Contraction time	0	0	-1	0
	Relaxation time	− 1	0	0	− 1
	Resting time	− 1	− 1	− 1	− 1
	Homogeneity	+ 1	0	+ 1	+ 1
RyR2 remodeling properties	PKA phosphorylation	0	0	0	0
	CaMKII phosphorylation	0	0	0	0
	RyR2 oxidation	0	0	0	0
	RyR2 S-nitrosylation	0	0	0	0
	Calstabin2 bound to RyR2	0	0	0	+ 1
Score	Total	0	− 1	+ 1	+ 2

The response of hiPSC-CMs to each drug is shown either as a positive response as + 1, negative response as − 1 or with no response as 0

### Propranolol, but not verapamil, tends to improve the contractile properties in RyR2-H29D hiPSC-CMs

**Fig. 3:**
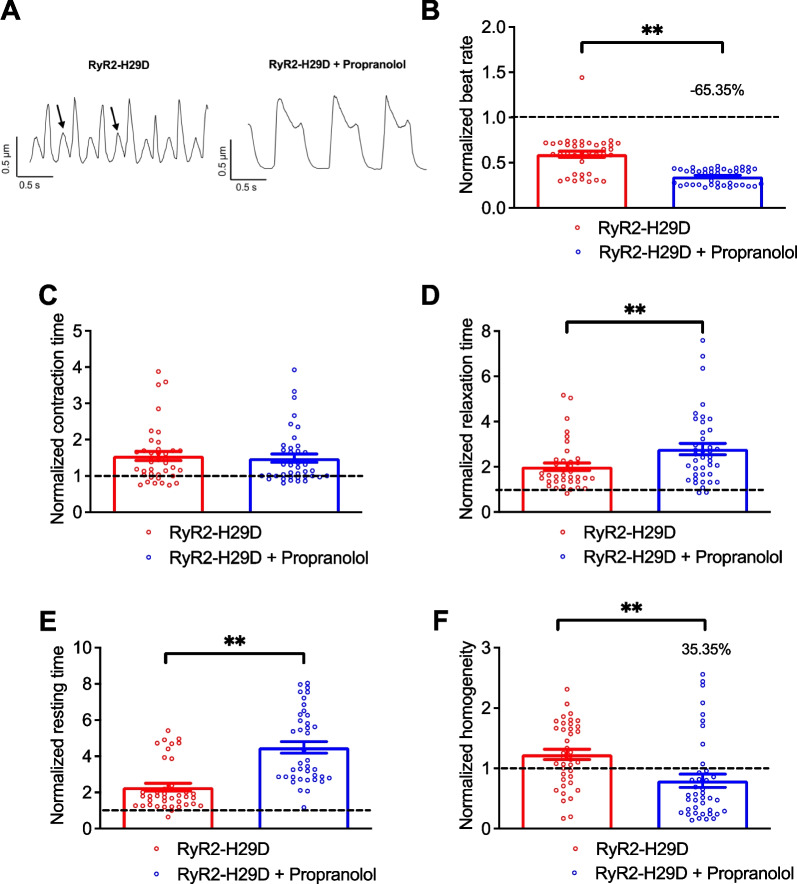
Propranolol improves the contractile properties in RyR2-H29D hiPSC-CMs. **A** Representative traces of contractile parameters in RyR2-H29D and RyR2-H29D hiPSC-CMs treated with 3 µM propranolol for 20 min. Aberrant contraction oscillations are marked with the arrows.** B** Normalized beat rate in RyR2-H29D hiPSC-CMs (red bars and red dots plot) and RyR2-H29D hiPSC-CMs treated with propranolol (blue bars and blue dots plot). **C** Normalized contraction time in RyR2-H29D hiPSC-CMs and RyR2-H29D hiPSC-CMs treated with propranolol. **D** Normalized relaxation time in RyR2-H29D hiPSC-CMs and RyR2-H29D hiPSC-CMs treated with propranolol.** E** Normalized resting time in RyR2-H29D hiPSC-CMs and RyR2-H29D hiPSC-CMs treated with propranolol. **F** Normalized homogeneity in RyR2-H29D hiPSC-CMs and RyR2-H29D hiPSC-CMs treated with propranolol. All parameters were normalized to the isogenic control (dotted line). The number of experiments is based on 3 independent biological replicates. The number of experiments varies from 39 to 40 videos for each scatter plot. Significance was calculated by Wilcoxon test. Data are presented as mean ± SEM. *, *p* < 0.05, **, *p* < 0.01

We previously demonstrated abnormal contractile properties in 2D cardiac sheets harboring the RyR2-H29D mutant channels. They were manifested by a decreased frequency and contraction time and a slower relaxation and resting time accompanied with a lower homogeneity compared to the isogenic control [7]. Thus, the RyR2-H29D cardiac sheets exhibited similar pathological features including lower beat rate and longer contraction/relaxation time (Additional file 1: Fig. S4A–D). The resting time was also longer in the RyR2-H29D cardiac sheets when compared to the isogenic control (Additional file 1: Fig. S4A and E). Moreover, we observed a better homogeneity factor for the isogenic control cardiac sheets (Additional file 1: Fig. S4F). We then tested the 4 drugs to evaluate their impact. In paired experiments, the same regions of spontaneous cardiac sheets were compared prior and upon drug treatment. We then normalized our different parameters to the isogenic control. We found that propranolol decreased the beat rate of RyR2-H29D hiPSC-CMs by 65.35% when compared to the isogenic control (0.59 ± 0.03 for the RyR2-H29D vs. 0.34 ± 0.01 for RyR2-H29D + propranolol, *p* < 0.01 in paired experiments) (Fig. 3A and B). No difference in average contraction time was observed (Fig. 3A and C). However, propranolol caused an increase in the normalized relaxation and resting time (Fig. 3A, D and E). As expected, propranolol caused a reduced beat rate of the isogenic control 2D cardiac sheets (Additional file 1: Fig. S5B). It caused longer contraction and relaxation times and increased the resting period (Additional file 1: Fig. S5B–D).

We then evaluated the homogeneity of contraction of the RyR2-H29D 2D cardiac sheets [7, 22]. The homogeneity of contraction was increased by 35.4% upon treatment with propranolol (1.23 ± 0.09 for the RyR2-H29D vs. 0.79 ± 0.10 for RyR2-H29D + propranolol, *p* < 0.01 in paired experiments) (Fig. 3A and F). Propranolol did not change the homogeneity of contraction of the isogenic control 2D cardiac sheets (Additional file 1: Fig. S5E).

**Fig. 4:**
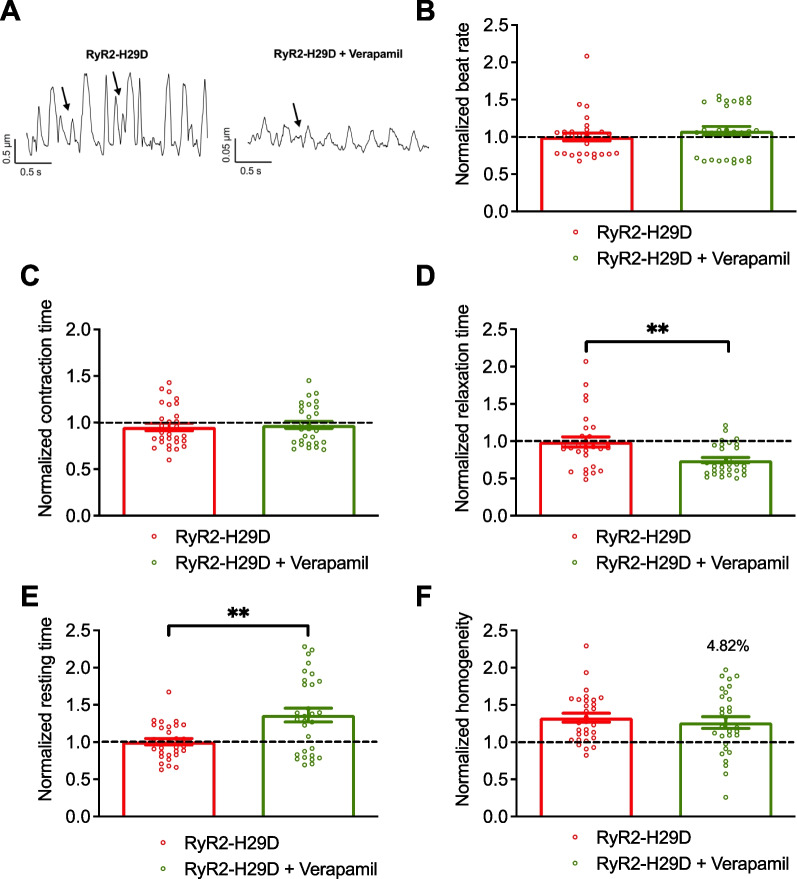
Verapamil does not prevent the aberrant contractile properties in RyR2-H29D hiPSC-CMs. **A** Representative traces of contractile parameters in RyR2-H29D hiPSC-CMs and RyR2-H29D hiPSC-CMs treated with 100 nM verapamil for 20 min. Aberrant contraction oscillations are marked with the arrows.** B** Normalized beat rate in RyR2-H29D hiPSC-CMs (red bars and red dots plot) and RyR2-H29D hiPSC-CMs treated with verapamil (green bars and green dots plot). **C** Normalized contraction time in RyR2-H29D and RyR2-H29D treated with verapamil.** D** Normalized relaxation time in RyR2-H29D and RyR2-H29D treated with verapamil. **E** Normalized resting time in RyR2-H29D and RyR2-H29D treated with verapamil. **F** Normalized homogeneity in RyR2-H29D and RyR2-H29D treated with verapamil. All parameters were normalized to the isogenic control (dotted line). The number of experiments is based on 3 independent biological replicates. The number of experiments varies from 28 to 30 videos for each scatter plot. Significance was calculated by Wilcoxon and paired t tests. Data are presented as mean ± SEM. **, *p* < 0.01

When applying verapamil, 16 out of 30 regions stopped beating in the RyR2-H29D monolayer cells, while this did not occur in the isogenic control group. In the 14 beating regions verapamil did not affect the beat rate or the normalized contraction time (Fig. 4A, B and C). Verapamil induced a decrease of the normalized relaxation time and an increase of the normalized resting time in RyR2-H29D monolayer cells, in agreement with the non-beating regions (1.00 ± 0.04 for the RyR2-H29D vs. 1.36 ± 0.09 for RyR2-H29D + verapamil, *p* < 0.01 in paired experiments) (Fig. 4A, D and E). There was no difference in the homogeneity of contraction upon treatment with verapamil (Fig. 4A and F). When applied on the isogenic control 2D cardiac sheets, verapamil did not affect the beat rate nor contraction time (Additional file 1: Fig. S6A and B). However, verapamil reduced the relaxation time (Additional file 1: Fig. S6C) while it increased the resting period (Additional file 1: Fig. S6D). Interestingly, verapamil decreased the contraction homogeneity of the isogenic control hiPSC-CMs (Additional file 1: Fig. S6E). Such a reduction in the contraction homogeneity is thought to be dysfunctional as we have seen before in RyR2-H29D 2D cardiac sheets [7]. A summary of the effects of propranolol and verapamil on the contractile properties is shown in Table 1.

### Flecainide and S107 prevent the aberrant contractile properties in RyR2-H29D hiPSC-CMs

**Fig. 5:**
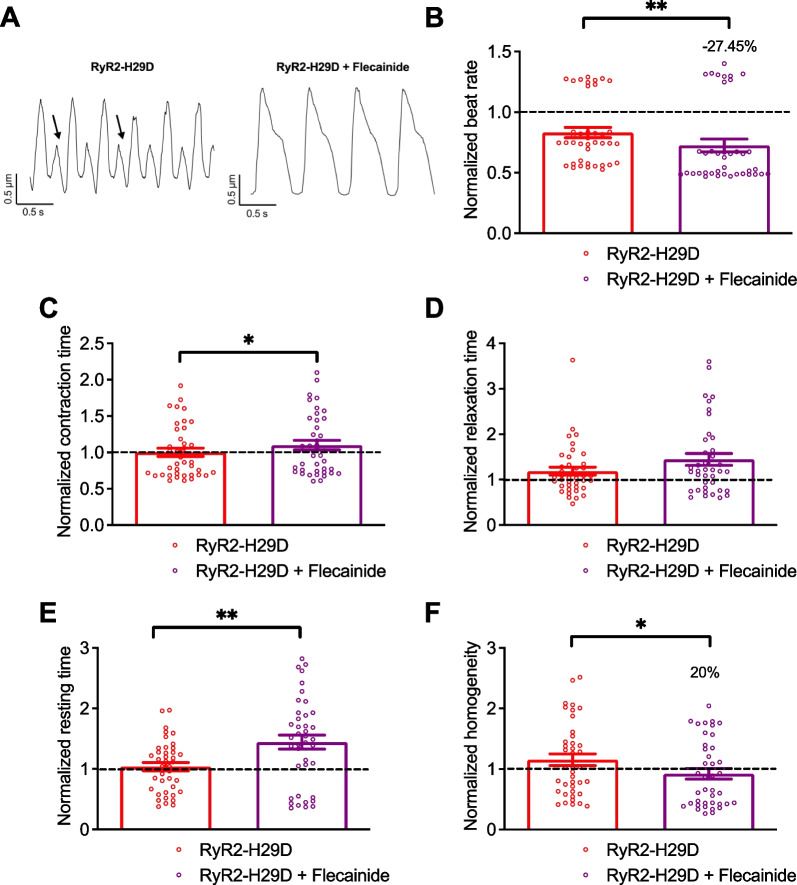
Flecainide is effective to prevent the aberrant contractile properties in RyR2-H29D hiPSC-CMs. **A** Representative traces of contractile parameters in RyR2-H29D and RyR2-H29D hiPSC-CMs treated with 5 µM flecainide for 20 min. Aberrant contraction oscillations are marked with the arrows.** B** Normalized beat rate in RyR2-H29D hiPSC-CMs (red bars and red dots plot) and RyR2-H29D hiPSC-CMs treated with flecainide (purple bars and purple dots plot). **C** Normalized contraction time in RyR2-H29D hiPSC-CMs and RyR2-H29D hiPSC-CMs treated with flecainide.** D** Normalized relaxation time in RyR2-H29D hiPSC-CMs and RyR2-H29D hiPSC-CMs treated with flecainide. **E** Normalized resting time in RyR2-H29D hiPSC-CMs and RyR2-H29D hiPSC-CMs treated with flecainide. **F** Normalized homogeneity in RyR2-H29D hiPSC-CMs and RyR2-H29D hiPSC-CMs treated with flecainide. All parameters were normalized to the isogenic control (dotted line). The number of experiments is based on 3 independent biological replicates. The number of experiments varies from 39 to 40 videos for each scatter plot. Significance was calculated by Wilcoxon and paired t tests. Data are presented as mean ± SEM. *, *p* < 0.05, **, *p* < 0.01

Application of 5 µM flecainide decreased the beat rate in RyR2-H29D cardiac sheets by 27.5% (0.83 ± 0.04 for the RyR2-H29D vs. 0.72 ± 0.05 for RyR2-H29D + flecainide, *p* < 0.01 paired experiments) (Fig. 5A and B). It caused an increase in the normalized contraction time while the normalized relaxation time remained unchanged (Fig. 5A, C and D). The normalized resting time increased in RyR2-H29D (1.04 ± 0.06 for the RyR2-H29D vs. 1.44 ± 0.11 for RyR2-H29D + flecainide, *p* < 0.01 paired experiments) (Fig. 5A and E). Interestingly, flecainide increased the homogeneity of contraction by 20% in RyR2-H29D cardiac sheets (Fig. 5A and F). Interestingly, the effect of flecainide was quite similar in the isogenic control hiPSC-CMs with a reduced beat rate (Additional file 1: Fig. S7A) and increased of the contraction (Additional file 1: Fig. S7B), relaxation and resting times (Additional file 1: Fig. S7C and D). Flecainide did not affect the contraction homogeneity (Additional file 1: Fig. S7E).

**Fig. 6:**
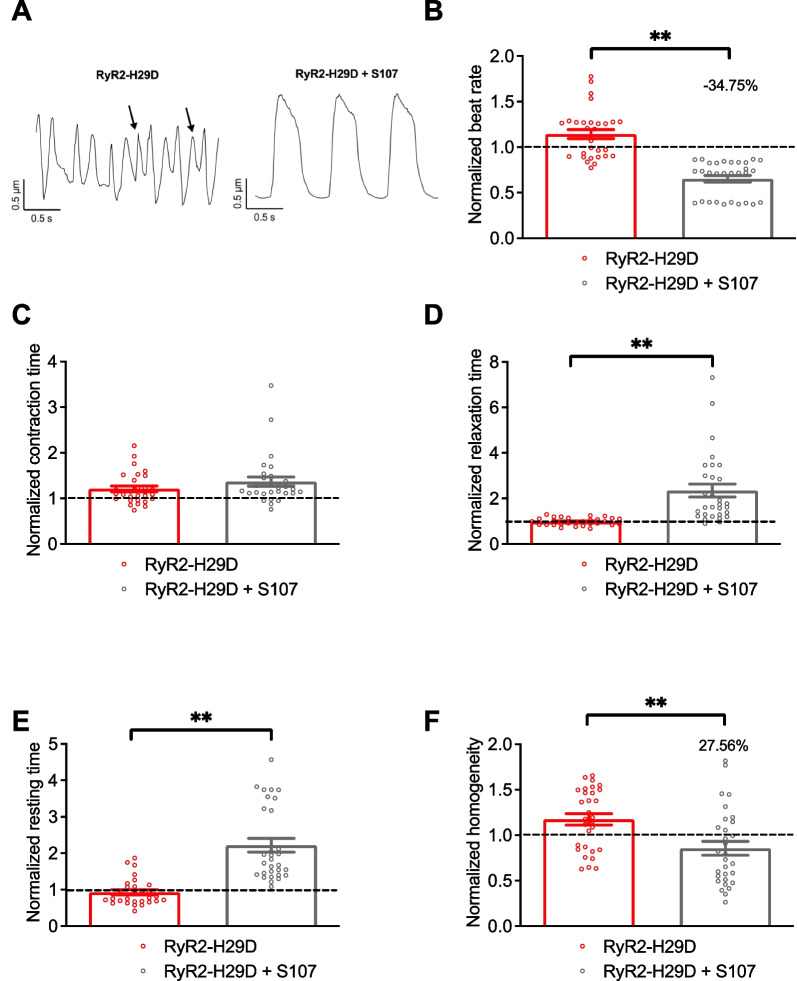
S107 is effective to prevent the aberrant contractile properties in RyR2-H29D hiPSC-CMs. **A** Representative traces of contractile parameters in RyR2-H29D hiPSC-CMs and RyR2-H29D hiPSC-CMs treated with 5 µM S107 for 20 min. Aberrant contraction oscillations are marked with the arrows. **B** Normalized beat rate in RyR2-H29D hiPSC-CMs (red bars and red dots plot) and RyR2-H29D hiPSC-CMs treated with S107 (gray bars and gray dots plot). **C** Normalized contraction time in RyR2-H29D hiPSC-CMs and RyR2-H29D hiPSC-CMs treated with S107. **D** Normalized relaxation time in RyR2-H29D hiPSC-CMs and RyR2-H29D hiPSC-CMs treated with S107. **E** Normalized resting time in RyR2-H29D hiPSC-CMs and RyR2-H29D hiPSC-CMs treated with S107. **F** Normalized homogeneity in RyR2-H29D hiPSC-CMs and RyR2-H29D hiPSC-CMs treated with S107. All parameters were normalized to the isogenic control (dotted line). The number of experiments is based on 3 independent biological replicates. The number of experiments varies from 26 to 30 videos for each scatter plot. Significance was calculated by Wilcoxon and paired t tests. Data are presented as mean ± SEM. **, *p* < 0.01

Application of 5 µM S107 also decreased the beat rate of the RyR2-H29D 2D cardiac sheets. We observed a change by 34.8% when compared to the isogenic control (1.14 ± 0.04 for the RyR2-H29D vs. 0.65 ± 0.03 for RyR2-H29D + S107, *p* < 0.01 paired experiments) (Fig. 6A and B). S107 did not affect the normalized contraction time (Fig. 6A and C). The normalized relaxation time as well as the normalized resting time increased upon S107 treatment (Fig. 6A, D and E). Importantly, S107 improved the contraction homogeneity by 27.6% in the RyR2-H29D 2D cardiac sheets (1.17 ± 0.06 for the RyR2-H29D vs. 0.85 ± 0.07 for RyR2-H29D + S107, *p* < 0.01 paired experiments) (Fig. 6A and F). Similar to flecainide, S107 reduced the beat rate and increased contraction, relaxation and resting times of the isogenic control 2D cardiac sheets (Additional file 1: Fig. S8A–D). S107 did not affect the contraction homogeneity of the isogenic control 2D cardiac sheets (Additional file 1: Fig. S8E). A summary of the effects of flecainide and S107 on the contractile properties is shown in Table 1.

## Discussion

We previously showed that the patient-derived CMs of a 32-year-old female proband harboring RyR2-H29D, who exhibited short-coupled PMVT, displayed aberrant electrical, Ca^2+^ handling and contractile properties leading to dysfunctional excitation–contraction coupling at rest. The aberrations were prevented by correcting this single nucleotide via CRISPR/Cas9 technology. This work, and our previous study using recombinant RyR2-H29D channels in HEK cells, revealed post-translational remodeling of the RyR2-H29D macromolecular complex associated with SR Ca^2+^ leak [6, 7]. Importantly, this established a link between an RyR2 gain-of-function mutation and the PMVT syndrome at rest. However, to date there is no specific treatment to prevent the recurrence of arrhythmias related to this clinical entity.

In the present work, we exploited the RyR2-H29D hiPSC-CMs and their isogenic control to perform a drug screening (summarized in Table 1) in an attempt at personalized medicine in vitro. We selected propranolol, verapamil, flecainide for their potency to prevent cardiac arrhythmias in patients [28–30] and S107 for its efficacy in preventing RyR2 Ca^2+^ leak experimentally [11–16]. We established a score for each drug according to its response to the different parameters summarized in Table 1. We found a higher score for S107 (score: + 2) as a potential drug to prevent the occurrence of short-coupled PMVT, followed by flecainide (score: + 1), propranolol (score: 0) and verapamil (score: − 1).

We distinguished drug applied on dissociated hiPSC-CMs and 2D cardiac sheets containing millions of connected hiPSC-CMs. Regarding the flecainide treatment, we picked 5 µM as we have shown its efficacy on CPVT hiPSC-CMs [11]. We applied it for 10 min for the RyR2 co-immunoprecipitation experiments and intracellular calcium imaging experiments on dissociated hiPSC-CMs. We applied it for longer time (20 min) on 2D cardiac sheets to reveal its impact on the contractile properties. Regarding the propranolol treatment, we picked 3 µM in agreement with the literature [31–33]. We applied propranolol at 3 µM for 10 min for the RyR2 co-immunoprecipitation and intracellular calcium imaging experiments and for 20 min on 2D cardiac sheets. For monitoring intracellular calcium handling, we employed a similar concentration of S107 (5 µM) previously applied in CPVT hiPSC-CMs for a duration of 10 min. Regarding the contractile properties under S107, we have shown in the past that 5 µM S107 overnight is effective to prevent the RyR2 Ca^2+^ leak in 3D cardiac embryoid bodies [11]. Given that embryoid bodies are large 3D structures with limited drug diffusion, we hypothesized that the same concentration (5 µM) could be applied and remain effective on 2D cardiac sheets. However, to align with the incubation time used for the other tested molecules in this study (flecainide, propranolol, and verapamil), we opted for a shorter incubation time of 20 min. Regarding the verapamil treatment, we used 10 nM on dissociated hiPSC-CMs and 100 nM on 2D cardiac sheets. We chose these concentrations based on the literature [34–36].

We previously demonstrated that the RyR2-H29D single-point mutation induces pathological post-translational modifications including PKA hyperphosphorylation at Ser2809, oxidation, S-nitrosylation and depletion of calstabin2, conferring a CPVT-like phenotype but under non-stress conditions [7]. In the present study, we first evaluated whether any of the 4 tested drugs could prevent these modifications. We found that none of the 4 drugs was able to prevent the PKA-phosphorylation, oxidation and S-nitrosylation. This observation suggests that these modifications cannot be reversed by short treatment (i.e., 10 min). Only S107 application was able to prevent the calstabin2 dissociation from the mutated RyR2 macromolecular complex to stabilize its closed state. These results confirmed our previous findings on the CPVT RyR2-D3638A mutation in patient-derived cardiomyocytes [11]. Of note, in our previous work focused on the characterization of RyR2-H29D hiPSC-CMs, we observed a similar signature with no PKA phosphorylation and no oxidation of RyR2 in the isogenic control hiPSC-CMs. These results are consistent with our previous findings in healthy RyR2 channels from human samples and WT animals, where we also observed the absence of PKA phosphorylation and oxidation [11, 13, 14, 37–39]. Therefore, our results on the isogenic control hiPSC-CMs are in line with our previously established data.

As calstabin2 depletion has been associated with RyR2 SR Ca^2+^ leak [6, 7, 40], we hypothesized that S107 not only prevents the depletion but also stabilizes the closed state conformation of RyR2 and prevents the Ca^2+^ leak. Indeed, when monitoring the intracellular Ca^2+^ handling, we found that S107 reduces both, the aberrant Ca^2+^ transients and diastolic leaky events that are associated with RyR2 leak [11]. When monitoring the contractile properties in 2D cardiac sheets, containing interacting RyR2-H29D hiPSC-CMs, we observed that S107 reduces the beat rate and prolongs the relaxation and resting times. S107 improves the spontaneous contraction homogeneity in RyR2-H29D hiPSC-CMs, similarly to what we previously found by correcting the RyR2-H29D mutation in the isogenic control hiPSC-CMs [7]. These results correlate with other studies showing beneficial effects of S107 in CPVT hiPSC-CMs [11, 15] as well as in mouse models of heart failure [16, 41]. The molecular mechanism allowing S107 to prevent calstabin2 depletion remains unclear. However, our recent structural findings on the skeletal muscle RyR1 channel indicate a binding site in the RY1&2 domain where ARM210 binds cooperatively with ATP and stabilizes the closed state of RyR1 [17]. As ARM210 and S107 belong to the same family of Rycal compounds, we could speculate that S107 acts similarly on RyR2 to prevent calstabin2 depletion.

We evaluated the effect of flecainide which was reported to be effective in CPVT patients, likely through Nav1.5 and possibly RyR2 current inhibition [11, 42]. In fact, the Knollman group observed that flecainide directly inhibits the RyR2 open probability by reducing the channel opening duration, especially under high luminal Ca^2+^ concentration. This group claimed that this mechanism is the principal action of flecainide to prevent CPVT. These results were challenged by some results showing that flecainide does not change the RyR2 channel kinetic and gating properties [10, 27, 43]. Therefore, the mechanism of action of flecainide remains controversial. Here, we found that flecainide only prevents the occurrence of aberrant Ca^2+^-transients but does not change the diastolic leak events via RyR2. These results are in agreement with our previous observations on CPVT hiPSC-CMs [11]. They suggest different mechanisms for flecainide and S107 to stabilize the SR Ca^2+^ handling in arrhythmic hiPSC-CMs at rest (PMVT) and under stress (CPVT) [43]. Regarding the contractile properties, we observed that flecainide further decreases the beat rate of the RyR2-H29D hiPSC-CMs. Hence, flecainide further exacerbates the bradycardia feature of RyR2-H29D hiPSC-CMs, when compared to isogenic control hiPSC-CMs. However, flecainide increases the contraction and relaxation time. Similar to S107, flecainide improves the contraction homogeneity in the RyR2-H29D hiPSC-CMs, which was previously shown crucial to improve the contractile properties of the RyR2-H29D hiPSC-CMs [7]. Our previous study demonstrated that flecainide partially prevents the SR calcium leak induced by the CPVT RyR2-D3638A mutant channels. Although flecainide was effective in preventing aberrant calcium transients, it did not abolish the diastolic leaky events induced by the RyR2 leaky channels [11]. Importantly, this partial effect of flecainide on SR calcium handling in CPVT aligns well with findings from another research group, which showed that flecainide does not modulate RyR2 channel properties in planar lipid bilayer experiments [43]. Additionally, it has been reported that flecainide has no apparent effect on isolated RyR2 channels incorporated into lipid bilayers under physiologically relevant conditions [44].

Regarding the specificity of flecainide, it is primarily recognized as a sodium channel blocker and is commonly used to treat ventricular arrhythmias. Although it may have some impact on intracellular calcium handling, its primary mechanism of action may not specifically target stabilizing RyR2 channels. This could be a potential reason why flecainide does not fully prevent the SR calcium leak caused by the RyR2-H29D mutation in our human PMVT model.

Propranolol treatment is able to reduce the aberrant Ca^2+^-transients and slow down the Ca^2+^ reuptake, mostly insured by the SERCA2a/phospholamban proteins. As a non-selective β-blocker, propranolol inhibits the β-adrenergic pathway that eventually modulates the PKA phosphorylation level of many protein targets, including SERCA2a and RyR2. We previously showed that beta-blockers reduced PKA-phosphorylation of RyR2 in human and canine models of heart failure and in CPVT hiPSC-CMs [11, 45, 46]. Surprisingly, propranolol does not affect the PKA-phosphorylation level of the RyR2-H29D mutant. Although the in vitro cell culture model does not fully reflect the in vivo adrenergic signaling, another mechanism may cause the PKA-phosphorylation of RyR2-H29D mutant channels. There may be a contact between the PKA-phosphorylation site at S2809 and H29D residues due to the subunit movements that we previously identified using 3D in-silico modeling [7]. Further experiments will be required to evaluate the impact of propranolol and β-blockade on RyR2-H29D hiPSC-CMs. We also observed that propranolol further decreases the beat rate of RyR2-H29D hiPSC-CMs, associated with an increase of the relaxation and resting times. Importantly, propranolol strongly improves the spontaneous contraction homogeneity in RyR2-H29D hiPSC-CMs, even more compared to what we observed by correcting the RyR2-H29D mutation in the isogenic control hiPSC-CMs. These results suggest that propranolol has a questionable impact in RyR2-H29D hiPSC-CMs. The present data correlate with other studies indicating that the β-blockade does not prevent the occurrence of arrhythmia in SC-TdP [9]. Moreover, it was reported that, despite the β-blockade, 5 out of 9 SC-TdP patients died suddenly [47]. Here, we propose that propranolol's inhibition of the cAMP-PKA signaling pathway leads to a reduction in the phosphorylation of phospholamban, which in turn relieves its inhibition on SERCA2a, the calcium pump responsible for SR Ca^2+^ reuptake during relaxation. As a result, the partial normalization of Ca^2+^ handling in PMVT hiPSC-CMs by propranolol contributes to a decrease in aberrant calcium transients and a slowdown in Ca^2+^ reuptake. This restoration of normal Ca^2+^ homeostasis ultimately leads to improved cardiomyocyte function. Furthermore, our observations indicate that propranolol treatment decreases the beat rate of RyR2-H29D hiPSC-CMs, accompanied by an increase in relaxation and resting times. The drug's ability to improve the spontaneous contraction homogeneity in RyR2-H29D hiPSC-CMs is noteworthy and appears to surpass the improvements observed when correcting the RyR2-H29D mutation in the isogenic control hiPSC-CMs. These findings provide valuable insights into propranolol's potential as a therapeutic agent for PMVT, as it not only targets aberrant calcium handling but also improves cardiomyocyte function and contraction homogeneity. The mechanisms underlying partial propranolol's effects on calcium handling in PMVT hiPSC-CMs warrant further investigation and may have significant clinical implications for patients with this condition.

In contrast with the 3 other drugs, verapamil was unable to prevent the aberrant SR Ca^2+^ handling caused by the RyR2-H29D mutation. As verapamil acts on DHPR, and based on our results, we speculate that verapamil is not a good candidate to prevent SR Ca^2+^ leak via RyR2 in the case of PMVT. Regarding the contractile properties, verapamil increases the relaxation time but slows the resting time in RyR2-H29D hiPSC-CMs. Importantly, in contrast with the 3 other tested drugs, verapamil does not recover the contraction homogeneity and fully inhibits almost half of the contracted areas in RyR2-H29D hiPSC-CMs. To compare, we did not observe this dramatic arrest in the isogenic control hiPSC-CMs. These results strongly suggest that the RyR2-H29D hiPSC-CMs are very sensitive to verapamil which does not appear to be appropriate to prevent both, the intracellular Ca^2+^ mishandling and contractile defects. It is possible that verapamil specifically slows down the spontaneous beat rate of RyR2-H29D hiPSC-CMs, which are known to exhibit abnormal reduced beat rate. Considering that patients with PMVT commonly experience bradycardia, the observed effect of verapamil in our study may be linked to the reduced spontaneous beat rate induced by the RyR2-H29D mutation. These results are consistent with the clinical observation of the proband with RyR2-H29D who continued to have PMVT despite verapamil therapy [6]. The sustained arrhythmias were suppressed only after catheter ablation was performed. Afterward, the patient treated with verapamil continued to have rare recurrences of non-sustained PMVT. The patient did not require escalation of therapy to include flecainide therapy. We believe that this was due to the success of catheter ablation of her PVCs as mentioned before [6]. If catheter ablation had not been performed or if it had not been successful, then flecainide would have been the next medication option.

Verapamil has been reported as an effective treatment for SC-TdP [9, 48]. It does not protect against the risk of SCD and implantable cardioverter-defibrillator (ICD) implantation [49, 50]. Catheter ablation of premature ventricular contractions triggering PMVT can be an effective adjunctive treatment for preventing the occurrence of future arrhythmias and ICD therapies [6]. Another study has shown that verapamil in combination with the β-blocker atenolol are potent to prevent SC-TdP in a patient carrying the RyR2-M995V mutation [51]. The consistency in our study of the clinical and molecular data with verapamil reinforces the personalized medicine that we attempted here.

Interestingly, the present study revealed that flecainide and verapamil worsened the SR Ca^2+^ handling in the isogenic control hiPSC-CMs manifested as increased diastolic leak events. These defects were not observed with S107 and propranolol. Verapamil even worsened the contraction homogeneity of isogenic control. In contrast, S107, flecainide and propranolol reduced the beat rate, increased the contraction/relaxation cycle time and improved the contraction homogeneity in isogenic control hiPSC-CMs. These results highlighted the complex link between the intracellular Ca^2+^ handling and ECC in hiPSC-CMs. Their spontaneous beating, likely associated with a Ca^2+^ clock involving RyR2 [52, 53], and their immaturity compared to adult ventricular CMs, may contribute to explain these ambivalent effects. As we [7] and others [54] have shown, the basic beat rate of control 2D cardiac sheets is closer to the one observed in fetal or neonatal cardiomyocytes, meaning closer to 150 bpm. In this context, we know that the spontaneous beat rate is higher than in adult heart. This is certainly a limitation of hiPSC-CM models derived from patients.

In the original study, we have shown that the isogenic control, harboring corrected RyR2-WT, exhibits control-like properties in action potentials, Ca^2+^ transients and contractile properties [7]. That is the reason why we have normalized the contractile properties of the drug-treated RyR2-H29D hiPSC-CMs to the ones of the isogenic control hiPSC-CMs. Furthermore, the ranking we provided does not consider the PMVT clinical features which could change the importance of the molecular and functional features we quantified under drug treatment.

## Conclusions

Using patient-specific hiPSC-CMs, we implemented a personalized medicine approach to testing candidate drug treatments in vitro. We found that S107, flecainide and propranolol were effective in reversing most of the abnormal features of RyR2-H29D hiPSC-CMs. In this context, S107 obtained the highest score, followed by flecainide and propranolol. We found that verapamil was ineffective in treating patient-derived CMs with short-coupled PMVT carrying the RyR2-H29D mutation in agreement with its clinical ineffectiveness to prevent PMVT in the patient. Our results highlight the increasing interest in patient-specific hiPSC-CMs, not only to model inherited diseases, but also to specify personalized medicine and rank candidate drugs for their efficacy. The ranking we found here might be different for another mutation causing PMVT. Such result would reinforce the interest in using patient-specific hiPSC-CMs for precision medicine. Our work supports previous studies on the development of hiPSC-based screening assays for testing individual drug reactions [55–57]. Altogether, they provide evidence that patient-specific hiPSC-CMs are a pertinent tool for drug screening and personalized medicine.

### Supplementary Information

**Additional file 1.** Supplemental data.

## Data Availability

All datasets used and/or analyzed during the current study are available from the corresponding author upon reasonable request.

## Abbreviations

PMVT: Polymorphic ventricular tachycardia
hiPSC: Human induced pluripotent stem cell
hiPSC-CM: Human induced pluripotent stem cell-derived cardiomyocyte
CM: Cardiomyocyte
Ca^2+^: Calcium
RyR: Ryanodine receptor
SR: Sarcoplasmic reticulum
ECC: Excitation–contraction coupling
PVC: Premature ventricular contraction
SC-TdP: Short-coupled torsade de pointes
SCD: Sudden cardiac death
PKA: Protein kinase A
CaMKII: Ca^2+^/calmodulin-dependent protein kinase II
ICD: Implantable cardioverter-defibrillator
