# Supplementary material for: Personalized medicine in the dish to prevent calcium leak associated with short-coupled polymorphic ventricular tachycardia in patient-derived cardiomyocytes
Source: Stem Cell Res Ther. 2023 Sep 23;14:266. doi: 10.1186/s13287-023-03502-5 (PMC10517551; doi:10.1186/s13287-023-03502-5)
Supplement: Supplementary file 1 — Additional file 1. Supplemental data. [file 13287_2023_3502_MOESM1_ESM.docx]

**Figure legends:**

**Figure S1. None of the drugs induce any RyR2 post-translational modifications in isogenic control hiPSC-CMs.** Immunoblots of the RyR2 co-immunoprecipitation showing the level of PKA and CaMKII-phosphorylation at Ser2809 (P2809) and Ser2815 (P2815) respectively, oxidation of cysteine (DNP), S-nitrosylation of cysteine (Cys-NO) and Calstabin2 (FKBP12.6) binding in treated and untreated isogenic control hiPSC-CMs with propranolol (3µM), verapamil (10nM), flecainide (5µM) and S107 (5µM). **(A)** Relative RyR2 PKA-phosphorylation level at Ser2809 in treated and untreated isogenic control hiPSC-CMs with propranolol, verapamil, flecainide and S107. **(B)** Relative RyR2 CaMKII-phosphorylation level at Ser2815 in treated and untreated isogenic control hiPSC-CMs. **(C)** Relative RyR2 oxidation level in treated and untreated isogenic control hiPSC-CMs. **(D)** Relative RyR2 S-nitrosylation level in treated and untreated isogenic control hiPSC-CMs. **(E)** Relative Calstabin2 amount bound to RyR2 in treated and untreated isogenic control hiPSC-CMs. The number of experiments is based on 3 independent biological replicates. Significance was calculated by Kruskal-Wallis test. Data are presented as mean ± SEM. IC=isogenic control and H29D=RyR2-H29D.

**Figure S2.** Full-length blots/gels of the immunoblots of the RyR2 co-immunoprecipitation showing the level of PKA and CaMKII-phosphorylation at Ser2809 (P2809) and Ser2815 (P2815) respectively, oxidation of cysteine (DNP), S-nitrosylation of cysteine (Cys-NO) and Calstabin2 (FKBP12.6) binding in treated and untreated isogenic control and PMVT hiPSC-CMs.

**Figure S3. Verapamil and flecainide induce aberrant Ca^2+^ leak in isogenic control hiPSC-CMs. (A)** Normalized Ca^2+^-transient amplitude in isogenic control (black bars and black dots plot), isogenic control treated with propranolol (3µM for 10 min) (blue bars and blue dots plot), verapamil (10nM for 10 min) (green bars and green dots plot), flecainide (5µM for 10 min) (purple bars and purple dots plot) and S107 (5µM for 10 min) (grey bars and grey dots plot) under 1Hz pacing (20 V and 5 ms duration). **(B)** Frequency of occurrence of aberrant Ca^2+^-transients in isogenic control, isogenic control treated with propranolol, verapamil, flecainide and S107. **(C)** Frequency of occurrence of diastolic leaky events in isogenic control, isogenic control hiPSC-CMs treated with propranolol, verapamil, flecainide and S107. **(D)** Rate of RyR2 Ca^2+^ release (dF/dt_max_ in ΔF/s) in isogenic control, isogenic control treated with propranolol, verapamil, flecainide and S107. **(E)** Decay time in isogenic control, isogenic control treated with propranolol, verapamil, flecainide and S107. The number of experiments is based on 3 independent biological replicates. The number of experiments varies from 25 to 237 cells for each scatter plot. Significance was calculated by Kruskal-Wallis test. Data are presented as mean ± SEM. *, p < 0.05, **, *p* < 0.01, ***, *p* < 0.001.

**Figure S4. Aberrant contractile properties in RyR2-H29D hiPSC-CMs. (A)** Representative traces of contractile parameters in RyR2-H29D and isogenic control hiPSC-CMs. Aberrant contraction oscillations are marked with the arrows. **(B)** Beat rate in RyR2-H29D hiPSC-CMs (red bars and red dots plot) and isogenic control hiPSC-CMs (black bars and black dots plot). **(C)** Average contraction time in RyR2-H29D hiPSC-CMs and isogenic control hiPSC-CMs. **(D)** Average relaxation time in RyR2-H29D hiPSC-CMs and isogenic control hiPSC-CMs. **(E)** Average resting time in RyR2-H29D hiPSC-CMs and isogenic control hiPSC-CMs. **(F)** Homogeneity in RyR2-H29D hiPSC-CMs and isogenic control hiPSC-CMs. The number of experiments is based on 3 independent biological replicates. The number of experiments varies from 60 to 69 videos for each scatter plot. Significance was calculated by Mann-Whitney test. Data are presented as mean ± SEM. *, p < 0.05, **, *p* < 0.01.

**Figure S5. Propranolol reduces the beat rate and increases the contraction/relaxation cycle time in isogenic control hiPSC-CMs. (A)** Beat rate in isogenic control hiPSC-CMs (black bars and black dots plot) and isogenic control hiPSC-CMs treated with 3µM propranolol for 20 min (blue bars and blue dots plot). **(B)** Average contraction time in isogenic control hiPSC-CMs and isogenic control hiPSC-CMs treated with propranolol. **(C)** Average relaxation time in isogenic control hiPSC-CMs and isogenic control hiPSC-CMs treated with propranolol. **(D)** Average resting time in isogenic control hiPSC-CMs and isogenic control hiPSC-CMs treated with propranolol. **(E)** Homogeneity in isogenic control hiPSC-CMs and isogenic control hiPSC-CMs treated with propranolol. The number of experiments is based on 3 independent biological replicates. The number of experiments varies from 26 to 30 videos for each scatter plot. Significance was calculated by Wilcoxon and Paired t tests. Data are presented as mean ± SEM. **, *p* < 0.01.

**Figure S6. Verapamil induces aberrant contractile properties in isogenic control hiPSC-CMs. (A)** Beat rate in isogenic control hiPSC-CMs (black bars and black dots plot) and isogenic control hiPSC-CMs treated with 100nM verapamil for 20 min (green bars and green dots plot). **(B)** Average contraction time in isogenic control and isogenic control treated with verapamil. **(C)** Average relaxation time in isogenic control and isogenic control treated with verapamil. **(D)** Average resting time in isogenic control and isogenic control treated with verapamil. **(E)** Homogeneity in isogenic control and isogenic control treated with verapamil. The number of experiments is based on 3 independent biological replicates. The number of experiments varies from 26 to 30 videos for each scatter plot. Significance was calculated by Wilcoxon and Paired t tests. Data are presented as mean ± SEM. **, *p* < 0.01.

**Figure S7. Flecainide reduces the beat rate and increases the contraction/relaxation cycle time in isogenic control hiPSC-CMs. (A)** Beat rate in isogenic control hiPSC-CMs (black bars and black dots plot) and isogenic control hiPSC-CMs treated with 5µM flecainide for 20 min (purple bars and purple dots plot). **(B)** Average contraction time in isogenic control hiPSC-CMs and isogenic control hiPSC-CMs treated with flecainide. **(C)** Average relaxation time in isogenic control hiPSC-CMs and isogenic control hiPSC-CMs treated with flecainide. **(D)** Average resting time in isogenic control hiPSC-CMs and isogenic control hiPSC-CMs treated with flecainide. **(E)** Homogeneity in isogenic control hiPSC-CMs and isogenic control hiPSC-CMs treated with flecainide. The number of experiments is based on 3 independent biological replicates. The number of experiments varies from 34 to 40 videos for each scatter plot. Significance was calculated by Wilcoxon and Paired t tests. Data are presented as mean ± SEM. **, *p* < 0.01.

**Figure S8. S107 reduces the beat rate and increases the contraction/relaxation cycle in isogenic control hiPSC-CMs. (A)** Beat rate in isogenic control hiPSC-CMs (black bars and black dots plot) and isogenic control hiPSC-CMs treated with 5µM S107 for 20 min (grey bars and grey dots plot). **(B)** Average contraction time in isogenic control hiPSC-CMs and isogenic control hiPSC-CMs treated with S107. **(C)** Average relaxation time in isogenic control hiPSC-CMs and isogenic control hiPSC-CMs treated with S107. **(D)** Average resting time in isogenic control hiPSC-CMs and isogenic control hiPSC-CMs treated with S107. **(E)** Homogeneity in isogenic control hiPSC-CMs and isogenic control hiPSC-CMs treated with S107. The number of experiments is based on 3 independent biological replicates. The number of experiments varies from 21 to 30 videos for each scatter plot. Significance was calculated by Wilcoxon and Paired t tests. Data are presented as mean ± SEM. *, p < 0.05, **, *p* < 0.01.

**Figures**

**Figure S1**

**Figure S2**

**Figure S3**

**Figure S4**

**Figure S5**

**Figure S6**

**Figure S7**

**Figure S8**
